# Exogenous Angiotensin I Metabolism in Aorta Isolated from Streptozotocin Treated Diabetic Rats

**DOI:** 10.1155/2016/4846819

**Published:** 2016-10-10

**Authors:** P. P. Wołkow, B. Bujak-Giżycka, J. Jawień, R. Olszanecki, J. Madej, J. Rutowski, R. Korbut

**Affiliations:** ^1^Department of Pharmacology, Jagiellonian University Medical College, Krakow, Poland; ^2^Center for Medical Genomics OMICRON, Jagiellonian University Medical College, Krakow, Poland; ^3^Department of Pharmacology, Medical Faculty, University of Rzeszów, Rzeszów, Poland

## Abstract

*Purpose*. Products of angiotensin (ANG) I metabolism may predispose to vascular complications of diabetes mellitus.* Methods*. Diabetes was induced with streptozotocin (75 mg/kg i.p.). Rat aorta fragments, isolated 4 weeks later, were pretreated with perindoprilat (3 *μ*M), thiorphan (3 *μ*M), or vehicle and incubated for 15 minutes with ANG I (1 *μ*M). Products of ANG I metabolism through classical (ANG II, ANG III, and ANG IV) and alternative (ANG (1–9), ANG (1–7), and ANG (1–5)) pathways were measured in the buffer, using liquid chromatography-mass spectrometry.* Results*. Incubation with ANG I resulted in higher concentration of ANG II (*P* = 0.02, vehicle pretreatment) and lower of ANG (1–9) (*P* = 0.048, perindoprilat pretreatment) in diabetes. Preference for the classical pathway is suggested by higher ANG III/ANG (1–7) ratios in vehicle (*P* = 0.03), perindoprilat (*P* = 0.02), and thiorphan pretreated (*P* = 0.02) diabetic rat. Within the classical pathway, ratios of ANG IV/ANG II (*P* = 0.01) and of ANG IV/ANG III (*P* = 0.049), but not of ANG III/ANG II are lower in diabetes.* Conclusions*. Diabetes in rats led to preference toward deleterious (ANG II, ANG III) over protective (ANG IV, ANG (1–9), and ANG (1–7)) ANG I metabolites.

## 1. Introduction

**Figure 1:**
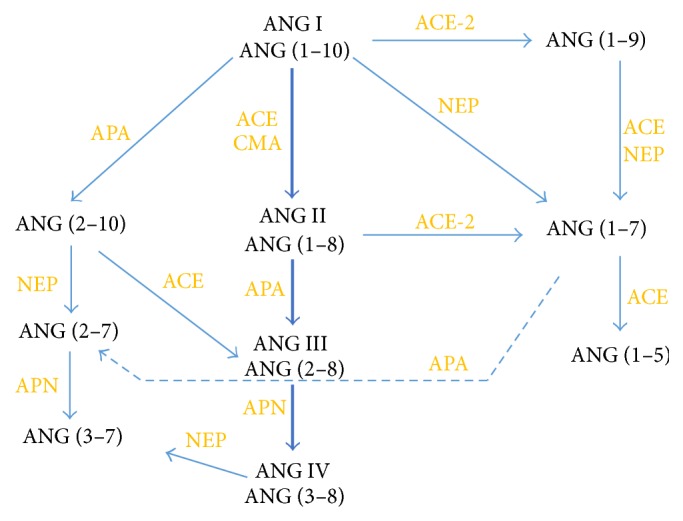
Main pathways of ANG I metabolism. ACE: angiotensin converting enzyme; ACE-2: angiotensin converting enzyme type 2; APA: aminopeptidase A; APN: aminopeptidase N; CMA: chymase; NEP: neutral endopeptidase.

Angiotensinogen metabolites are involved in vascular complications of diabetes, such as myocardial infarction, stroke, nephropathy, retinopathy, and foot ulcers [1]. In these complications, angiotensin II (ANG II) is the most extensively studied metabolite. However, other products of metabolism also deserve attention (see Figure 1). An alternative pathway, through angiotensin (1–9) [ANG (1–9)], angiotensin (1–7) [ANG (1–7)], and angiotensin (1–5) [ANG (1–5)], sparked interest because ANG (1–7) and ANG II have contrasting effects on vasculature [2]. A classical pathway, in addition to ANG II, contains also angiotensin III (ANG III) and angiotensin IV (ANG IV). Effects of ANG IV in diabetes may also differ from those of ANG II and ANG III [3].

Our intention was to compare concentration of more established angiotensin I (ANG I) metabolites, produced by a large artery of diabetic and control rats. We used an established model of diabetes induction* in vivo*, by a single injection of streptozotocin (STZ), a toxic chemical, preferentially transported to and directed against insulin producing, pancreatic beta cells from islets of Langerhans [4]. Destruction of beta cells by STZ causes an absolute deficiency of insulin, which resembles type 1 diabetes mellitus in men, although without involvement of the immune system as an executor of destruction. Single injection of STZ leads within a few days to a statistically significant hyperglycemia. We chose not to treat rats with insulin. This leads within a few weeks to symptoms of acute, insulin-deficient diabetes, with significant loss of body weight in all STZ injected rats. We expected that by the time when animals were hyperglycemic and visibly sick, pathways of ANG I metabolism were already altered.

Fragments of aorta, isolated and suspended in perfusion buffer, provide a tool to study ANG I metabolism in a single organ, without interference from other metabolically active sites, such as kidney or heart. Rat aorta was previously used as a model of diabetic vascular dysfunction [5]. However, metabolism of endogenous angiotensins was shown to be similar in diabetic and control animals [6]. Therefore, we used exogenous ANG I at a relatively high, compared to endogenous, concentration. Our intention was to saturate enzyme capacity to process substrates and to study the differences between the diabetic and control rats under these conditions. Our* working hypothesis* was that concentration of metabolites of exogenous ANG I was different in diabetic compared to control rats. We were especially interested to study whether classical (ANG II, ANG III, and ANG IV) and alternative (ANG (1–9), ANG (1–7), and ANG (1–5)) metabolic pathways were similarly active and whether ratios between individual components of a given pathway did not differ between the studied groups.

Several groups have previously demonstrated dysregulation of the angiotensin metabolic and signaling pathway in the aorta of STZ-treated, diabetic rats, for example, upregulation of angiotensin II type 1 receptor expression [7] and hypercontractility of aortic rings in response to ANG II [8]. Members of the alternative pathway of ANG I metabolism also appear to play an important role in STZ-treated, diabetic rats, because activation of angiotensin converting enzyme 2 improves endothelial function [9]. This makes the study of ANG I metabolism in this context an interesting research goal.

## 2. Materials and Methods

### 2.1. Animals

Animal experiments were approved by an Ethical Committee of the Jagiellonian University (number 12/2006). Three-month-old Sprague-Dawley rats were divided into two groups: a control group (CTRL, *n* = 7), injected with vehicle (citrate buffer), and diabetic group, injected intraperitoneally with streptozotocin (STZ, 75 mg/kg, *n* = 8) in citrate buffer (pH = 4.5). Animals were kept under controlled conditions of constant room temperature and humidity, with 12/12 h light/dark cycle, and a free access to food and water. Blood glucose levels were checked 7 days after STZ administration. The experiment lasted 4 weeks. After this time, blood glucose concentration and body weight were measured in both groups of animals. Animals were given fraxiparine (2850 IU, i.p.) and anaesthetized with 50 mg of thiopentone (50 mg/mL, i.p.). Fragments of aorta were excised through abdominal incision.

### 2.2. Preparation of Tissue Fragments and “Organ-Bath” Procedure

Tissue fragments were washed with cold, standard Krebs-Henseleit solution and cleaned of thrombi and tissue remnants. Blood vessels were cut into a suitable number of rings and opened flat. Tissue incubation was performed as described previously [10]. Briefly, tissue fragments were incubated in triplicate in 400 *μ*L of freshly prepared, bubbled Krebs buffer (at 37°C) with or without angiotensin converting enzyme (ACE) inhibitor, perindoprilat, or neutral endopeptidase (NEP) inhibitor, thiorphan (with limited, nonspecific ACE inhibitory properties), both at final concentration of 3 *μ*M. After 5 minutes of pretreatment, ANG I was added to a final concentration of 1 *μ*M and samples were incubated for further 15 minutes. Subsequently, incubation buffer was removed and analyzed by LC/MS. Tissue samples were dried at 60°C and weighed to estimate peptide production per mg of dry tissue.

### 2.3. LC/MS Measurement of Angiotensin Peptides

Metabolites of ANG I were analyzed by liquid chromatography-mass spectrometry (LC/MS) method, developed in our previous studies [10, 11], with analytical conditions optimized to current type of samples. Separation of peptides was performed on a reversed-phase HPLC system, equipped with a quaternary high pressure pump L-7000 (Merck, Germany), using a Purospher STAR RP C18e column (125 mm × 2 mm ID, 5 *μ*m particle size) with an appropriate guard C18 column (4 mm × 4 mm ID, 5 *μ*m particle size). Samples were injected onto chromatographic column in a volume of 50 *μ*L. The optimized mobile phase solvents were 5% acetonitrile in a buffer of 4 mM ammonium formate with 4 mM of formic acid (phase A) and 90% acetonitrile in the same buffer (phase B). Angiotensin peptides were separated at a flow rate of 0.25 mL/min with a linear gradient of B in A.

Mass spectrometric detection was performed using an LCQ ion-trap mass spectrometer (Finnigan, San Jose, USA) with an electrospray (ESI) source. All experiments were carried out in the positive ion mode (ion spray voltage 5 kV; capillary voltage 46 V; capillary temperature 200°C; nitrogen flow rate 65 psi) and selective ion monitoring (SIM) mode;* m/z* values of monitored ions were the following: 450 for ANG (1–7) (MW = 899,02), 466 for ANG III (MW = 931,11), 524 for ANG II (MW = 1046,19), 592 for ANG (1–9) (MW = 1183,34), 649 for ANG I (1296,51), 665 for ANG (1–5) (664,76), and 775 for ANG IV (774,92). Acquired data were analyzed by Xcalibur Software v. 1.2.

Samples for calibration curves of each examined peptide (mixture of standards) were analyzed in the same mode as above. Concentrations of ANG I metabolites were calculated using the standard calibration curves. They were constructed by linear regression analysis with plotting of peak area versus angiotensin concentration. Calibration curves were prepared for each examined peptide at a concentration range of 20 pM–100 nM.

### 2.4. Chemicals

STZ, thiorphan, ANG II, and ANG (1–7) were purchased from Sigma Chemicals (USA). ANG I, ANG III, ANG IV, ANG (1–9), and ANG (1–5) were purchased from Bachem (USA). Perindoprilat was a gift from Servier (France). Formic acid (99%), trifluoroacetic acid (TFA), and ammonium formate were purchased from Fluka (USA). Acetonitrile (J. T. Baker, USA) and water (Rathburn, Scotland) were of HPLC grade.

### 2.5. Statistics

Concentrations of angiotensins were expressed as in pmol/mg dry tissue. All values in the text, tables, and figures are expressed as median [25th, 75th percentile]. Studied groups were compared with nonparametric Kruskal-Wallis. *P* values of less than 0.05 were considered statistically significant.

## 3. Results

For the experiments, we used 3-month-old Sprague-Dawley rats, with median [25th and 75th percentile] 328 [292; 355] g body weight. Four weeks after STZ injection, body weights of STZ (*n* = 8) and CTRL (*n* = 7) rats were 201 [193; 223] g and 356 [330; 365] g, respectively. All animals in STZ group developed diabetes with median [25th; 75th percentile] blood glucose level of 23.56 [20.28; 25.94] mmol/L, while in the CTRL group it was 6.78 [5.97; 7.56] mmol/L.

**Figure 2:**
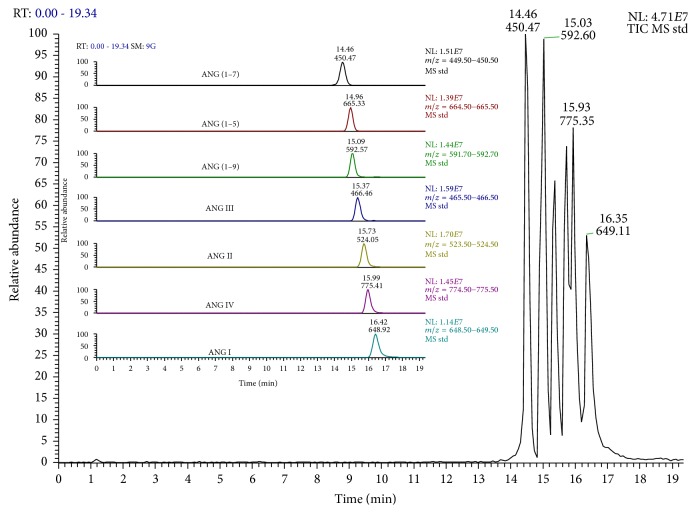
Representative chromatogram of angiotensins standards mixture. Insert: chromatograms of extracted monitored ions of each analyzed angiotensin.

**Figure 3:**
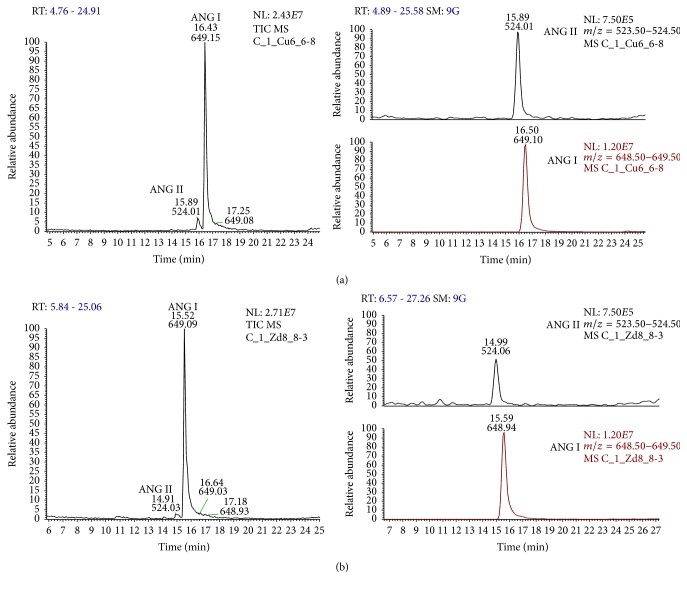
Representative chromatograms of products of ANG I conversion by aorta of STZ rats (a) and control rats (b). Left panel: TIC chromatogram (peaks represent relative abundance, 100% = ANG I); right panel: extracted chromatograms for monitored ions of ANG II and ANG I (unified scale).

The weight of aorta fragments used for experiments did not differ between the study groups (0.83 ± 0.23 mg for CTRL versus 0.76 ± 0.21 mg for STZ rats, *P* = 0.56). We compared vascular metabolism of exogenous ANG I (added to the perfusion buffer to the final concentration of 1 *μ*M) in STZ and CTRL rats. Representative chromatogram of extracted monitored ions of each analyzed angiotensin is shown in Figure 2. Representative chromatograms of products of ANG I conversion by aorta of STZ-treated and control rats are shown in Figures 3(a) and 3(b), respectively. The concentrations of the products of the classical (ANG II, ANG III, and ANG IV) and alternative [ANG (1–9), ANG (1–7), and ANG (1–5)] pathways of ANG I metabolism, measured in perfusion buffer, are presented in Table 1.

The data show that only the concentration of ANG II, but not of any other ANG I metabolite, is significantly higher in STZ group of hyperglycemic rats (*P* = 0.02). Increased concentration of ANG II was previously demonstrated to adversely impact many comorbidities (e.g., hypertension) and complications (e.g., ischaemic heart disease, nephropathy) of diabetes.

Exogenously added ANG I is metabolized mainly by one of three enzymes: through either ACE to ANG II or ACE2 to ANG (1–9) or NEP to ANG (1–7). The possibility of ANG I metabolism through chymase or other peptidases cannot be ruled out. We therefore studied whether enzyme inhibitors differently affect ANG I metabolism of STZ versus CTRL rats. We used inhibitor of angiotensin converting enzyme (ACE) perindoprilat (final concentration 3 *μ*M) and thiorphan (final concentration 3 *μ*M), preferential inhibitor of neutral endopeptidase (NEP), with limited nonspecific ACE inhibitory activity.

**Figure 4:**
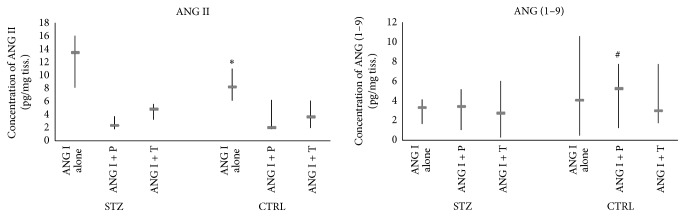
Concentrations (median, minimum, and maximum) of ANG II and ANG (1–9), produced from exogenously added ANG I, by the aortic rings of STZ and CTRL rats, pretreated with perindoprilat (ANG I + P) or thiorphan (ANG I + T). ^*∗*^ *P* < 0.05, ANG II concentration was higher in (ANG I only) group of STZ aorta. ^#^ *P* < 0.05, ANG (1–9) concentration was higher in (ANG I + P) group of CTRL aorta.

The effect of perindoprilat on ANG I metabolism is compared between both studied groups and presented in Table 2. The data show that, in STZ group, pretreatment with perindoprilat results in lower ANG (1–9) concentration after incubation with ANG I than in CTRL group (*P* = 0.048). This suggests that, in diabetic rats, perindoprilat uncovers more efficient metabolism of ANG I through a classical pathway. Such suggestion is further enhanced by the observation that use of thiorphan, an inhibitor of NEP, which is also to a certain degree a nonspecific ACE inhibitor, results in borderline higher concentration of ANG I in STZ rats (*P* = 0.06) (Table 3, Figure 4). ANG I, remaining at the higher concentration in the perfusion buffer from STZ rats, could result either from more efficient ACE or less efficient ACE2 activity in diabetic rats. Borderline lower concentration of ANG I in the buffer from thiorphan-pretreated aorta of CTRL rats, without visible concomitant increase in measured ANG I metabolites, may suggest that in this situation ANG I, rather than being used by classical or alternative pathways, is converted to some other metabolites, not measured by us (see Figure 1). For example, aminopeptidase A activity with conversion to ANG (2–10) could become more prominent in such case.

Given the suggestions that ACE2-initiated alternative pathway of ANG I metabolism may be less active in STZ-treated rats, to confirm preferential use of classical over alternative pathway of ANG I metabolism in STZ-treated rats, we compared the ratios of key metabolites of both pathways in the studied groups. The data for ANG II/ANG (1–9), ANG II/ANG (1–7), ANG III/ANG (1–9), ANG III/ANG (1–7), ANG IV/ANG (1–9), and ANG IV/ANG (1–7) ratios in STZ-treated and CTRL rats without pretreatment and after perindoprilat or thiorphan pretreatment are presented in Table 4.

The data show that ANG III/ANG (1–7) ratio is increased in nonpretreated diabetic rats while ANG III/ANG (1–7), ANG IV/ANG (1–9), and ANG IV/ANG (1–7) ratios are all increased in diabetic rats after perindoprilat pretreatment. Similarly, thiorphan pretreatment results in increased ANG III/ANG (1–7) ratio in diabetic rats. These data further demonstrate that the classical pathway of ANG I metabolism is preferred over an alternative pathway in rats with STZ-induced diabetes, compared to normoglycemic rats.

Finally, since the classical pathway of ANG I metabolism is used preferentially over an alternative pathway in STZ-treated rats in comparison to citrate buffer-treated CTRL animals, we wanted to study whether all components of the classical pathway are equally preferred by the STZ-treated animals. To test this, we compared the ANG IV/ANG II, ANG IV/ANG III, and ANG III/ANG II ratios between both studied groups (Table 5).

The data presented in Table 5 surprisingly show that the ratios of ANG IV to both ANG II and ANG III are significantly lower in STZ-treated than in CTRL animals. In contrast, ratio of ANG III/ANG II does not differ between the studied groups. Therefore, this demonstrates that components of the classical pathway of ANG I metabolism, which is preferentially used over an alternative pathway in hyperglycemic STZ-treated rats, are not equally preferred.

ANG IV appears to be less likely a product of the aorta metabolism than ANG II or ANG III in STZ-treated rats, after incubation with ANG I. This may have important implications for vascular complications of diabetes, because ANG IV plays a different role in vasculature, compared to ANG II or ANG III (described in detail in the Discussion). It is not known whether lower ANG IV/ANG II and ANG IV/ANG III ratios in STZ group result, for example, from lower ANG IV synthesis, faster ANG IV metabolism, or other reasons. Lower ANG IV/ANG II ratio in STZ group could be potentially explained simply by higher denominator in this ratio, due to, previously shown in Table 1, higher concentration of ANG II in these animals. However, such reasoning is not sufficient to explain lower ANG IV/ANG III ratio in hyperglycemic rats.

## 4. Discussion

Toxicity of streptozotocin against beta cells from Langerhans islets of pancreas leads to an absolute insulin deficiency and chronic hyperglycemia in experimental animals, similar to symptoms observed in patients with type 1 diabetes mellitus. Persistent hyperglycemia causes endothelial dysfunction, an important contributor to vascular complications of diabetes [12], for example, myocardial infarction, retinopathy, or nephropathy. Pathomechanisms of diabetic complications, which lead to increased patient mortality and lower quality of life, are known only partially.

Angiotensin II is an endogenous peptide with very potent vascular effects, including vasoconstriction and stimulation of thrombosis. It is the best studied member of the “classical” renin-angiotensin system (RAS), frequently implicated in pathogenesis of diabetic complications [1]. The role of ANG II was confirmed by clinical trials, in which inhibitors of ANG II formation or signaling significantly reduced the incidence of vascular complications in DM patients [13, 14]. These improvements appear to result from protective actions on skeletal muscle [15] and pancreatic islets [16], enhanced insulin sensitivity associated with decreased adipocyte size [17], and increased transcapillary glucose transport [18]. Conversely, ANG II can cause insulin resistance by interfering with the insulin-stimulated insulin receptor signaling [19].

Recent research revealed, however, that angiotensin metabolites comprise much wider spectrum of active, endogenous substances, with less uniform vascular effects than previously expected. For example, a member of the so-called “alternative” pathway of angiotensin metabolism, ANG (1–7), acts through a distinct receptor, which leads to vasodilation when activated [20]. ANG I is the common precursor of both “classical” and “alternative” metabolic pathways. The objective of our study was to compare, in a comprehensive manner, concentration of angiotensin metabolites, produced by aorta of diabetic and control rats.

We used a well-described model of diabetes in rat, induced by a single injection of streptozotocin, a toxic agent preferentially transported to beta cells through GLUT2 glucose transporter [21]. Our* working hypothesis* was that diabetes led to changes in aortic angiotensin metabolism, reflected by differences in the final concentration of metabolites in the incubation buffer. We added exogenous ANG I, a substrate for both classical and alternative pathways of metabolism of angiotensin peptides. High concentration of exogenous ANG I used and large amount of ANG I remaining in the buffer after incubation ensure that, under these experimental conditions, enzymatic capacity to process angiotensins by aorta fragments was saturated. Such “stress-test” conditions provide better chance to unravel changes of metabolism during diabetes than low-level baseline metabolism.

Our results showed that concentration of ANG II is higher after incubation with aorta of diabetic rat. It is not known whether this is due to higher production of ANG II from ANG I or lower metabolism of ANG II to downstream products. We cannot exclude the possibility that diabetes changed expression of ACE, ACE2, NEP, or other enzymes that affect ANG II formation or decomposition. Perindoprilat, inhibitor of ACE, should redirect ANG I metabolism towards an “alternative” pathway, leading to increased ANG (1–9) and ANG (1–7) concentrations. However, our results suggest that the “alternative” pathway is less active in diabetic rat aorta, because concentration of ANG (1–9) after perindoprilat is lower in this group. This is further confirmed by the fact that ANG III/ANG (1–7) ratio is higher in aorta from diabetic rat, both without pretreatment and after perindoprilat or thiorphan pretreatment. Interestingly, the preference for “classical” pathway of ANG I metabolism in diabetic rat aorta is limited to ANG II and ANG III but does not include ANG IV, which may play a different role. Our data show that the ratios of ANG IV/ANG II and ANG IV/ANG III, but not of ANG III/ANG II, are lower in diabetes.

In summary, our data show that in aorta of diabetic rat the classical pathway of exogenous ANG I metabolism to ANG II and ANG III dominates over an alternative pathway. Interestingly, basal plasma and aortic content of ANG II is not modified in diabetes, induced by streptozotocin [6]. The effect of ANG II on contractility of aortic rings in diabetes is uncertain. According to some researchers, it remains unchanged [22], especially in early diabetes [23]. Others show increased contractility in response to ANG II later in diabetes or in high glucose environment [24]. Such increased contractility could be only partially prevented by ACE inhibitor [8] although ACE inhibitors could effectively normalize ACE activity [25] and oxidative stress [26]. Therefore, other angiotensin metabolites may play a role in aortic ring hypercontractility.

The alternative pathway of metabolism of ANG I to ANG (1–9) and ANG (1–7) was less used than the classical pathway in our experimental model of diabetic rat aorta. Mahmood et al. [27] demonstrated unaltered concentration of ANG II and ANG (1–7) but lower of ANG (1–9) in perfusate from diabetic rat heart. Activation of ACE2, which converts ANG I to ANG (1–9) and ANG II to ANG (1–7), improves endothelial function [9], for example, and prevents diabetic retinopathy [28]. In our model, lower concentration of ANG (1–9) after perindoprilat pretreatment and higher ANG III/ANG (1–7) ratios can be potentially explained by lower ACE2 expression in diabetic rat aorta. Indeed, although increased ACE2 expression was observed by other researchers in early diabetes, the expression of this enzyme was lower later during the disease [29]. Studying an effect of ACE2 inhibitors in this experimental setting could allow better understanding of alternative pathway of ANG I metabolism in diabetes. Lack of use of inhibitors of potentially important enzymes (e.g., ACE2, chymase, aminopeptidase A, and aminopeptidase N) is a limitation of our study.

ANG IV appears to be used in our experimental model differently than other members of the classical pathway of angiotensin metabolism. Although concentrations of ANG IV are not modified directly by diabetes, with and without drug pretreatment, ratios of ANG IV to ANG II and ANG III are lower in diabetic rat aorta. Although a higher concentration of ANG II in STZ-treated group could potentially explain drop in ANG IV/ANG II ratio, this cannot suffice in case of the observed ANG IV/ANGIII ratio drop. Since our study observed an effect of perindoprilat on ANG IV at the level of ratios only, but not directly on concentration of this metabolite, we admit that more convincing results could be potentially seen if aortic rings were stimulated with, for example, ANG III.

An important pioneer step forward to understand the role of ANG IV in vasculature was made recently with the report by Vinh et al. that chronic ANG IV treatment reverses endothelial dysfunction in apoE-deficient mice fed a high fat diet [30]. The same group further evidenced that, in this experimental model of atherosclerosis, ANG IV treatment was able to restore endothelial function, even when administered in mice with advanced atheroma [31]. The objective of the study performed by Nasser et al. was to examine whether ANG IV plays a similar protective role against endothelial dysfunction and vascular hypertrophy in the mice model of STZ-induced diabetes [32]. This study provided evidence that AT2 and AT4 receptors have opposite effects on diabetes-induced vascular alterations. Chronic ANG IV treatment restored endothelium-dependent relaxation, blunted thickening of the aortic media, and reversed diabetes-induced changes in production of reactive oxygen species.

Although clinical studies suggest that RAS blockade may prevent, delay, or ameliorate diabetes complications, the mechanism remains uncertain. Apart from well-known beneficial effects of ANG II signaling inhibition and recently suggested beneficial effects of ANG (1–7) signaling augmentation, ANG IV emerged as yet another interesting component of the RAS system in diabetes. This offers new opportunities for examining physiological roles of ANG IV in the fields of cardiovascular metabolism and in pathophysiological conditions like diabetes and hypertension. ANG IV analogs and AT4 agonists may provide new therapeutic options for better vascular prevention and therapy in diabetic patients. Still new recognition sites may be unveiled for this and other angiotensin fragments [33].

## 5. Conclusions

- ANG II concentration was higher in organ bath of diabetic rat aorta, incubated with ANG I, than in control rat aorta.
- The alternative pathway of metabolism of ANG I to ANG (1–9) and ANG (1–7) was less used than the classical pathway in this experimental model.
- Ratios of protective ANG IV to deleterious ANG II or ANG III are lower in diabetic rat aorta.

## Figures and Tables

**Table 1:** Concentrations of the products of metabolism of exogenously added (to a final concentration of 1 *µ*M) angiotensin I by the aorta fragments of streptozotocin and saline treated animals (pg/mg of dry tissue; median [25th, 75th percentile]). *P* values are from Kruskal-Wallis nonparametric tests.

Analyte	Concentration in streptozotocin treated rats (*n* = 8), pg/mg of dry tissue; median [25th, 75th percentile]	Concentration in control rats (*n* = 7), pg/mg of dry tissue; median [25th, 75th percentile]	*P* value
Angiotensin I	773.41 [667.48, 877.49]	690.10 [546.29, 825.94]	0.42
Angiotensin II	13.50 [10.46, 14.89]	8.27 [7.47, 10.75]	**0.02**
Angiotensin III	3.80 [3.34, 4.89]	3.31 [2.63, 3.72]	0.20
Angiotensin IV	1.14 [0.84, 1.53]	1.60 [1.13, 1.73]	0.25
Angiotensin (1–9)	3.37 [1.94, 4.15]	4.12 [1.32, 5.10]	0.41
Angiotensin (1–7)	2.39 [1.81, 3.14]	2.20 [1.92, 3.21]	0.82
Angiotensin (1–5)	1.06 [0.86, 1.72]	1.22 [0.77, 1.95]	0.91

**Table 2:** Concentrations of the products of metabolism of exogenously added (to a final concentration of 1 *µ*M) angiotensin I by the aorta fragments pretreated with perindoprilat (final concentration 3 *µ*M) from streptozotocin and saline treated animals (pg/mg of dry tissue; median [25th, 75th percentile]). *P* values are from Kruskal-Wallis nonparametric tests.

Analyte	Concentration in streptozotocin treated rats (*n* = 8), pg/mg of dry tissue; median [25th, 75th percentile]	Concentration in control rats (*n* = 7), pg/mg of dry tissue; median [25th, 75th percentile]	*P* value
Angiotensin I	941.9 [587.3, 1205.7]	884.1 [689.1, 975.3]	0.95
Angiotensin II	2.42 [2.21, 3.07]	2.11 [1.81, 2.59]	0.18
Angiotensin III	3.25 [2.55, 4.07]	2.45 [2.29, 3.85]	0.41
Angiotensin IV	1.10 [0.84, 1.49]	0.79 [0.62, 1.31]	0.18
Angiotensin (1–9)	3.48 [1.69, 4.25]	5.29 [3.65, 7.47]	**0.048**
Angiotensin (1–7)	2.16 [1.95, 3.28]	2.57 [2.38, 3.63]	0.22
Angiotensin (1–5)	1.19 [0.80, 1.50]	0.95 [0.80, 1.17]	0.65

**Table 3:** Concentrations of the products of metabolism of exogenously added (to a final concentration of 1 *µ*M) angiotensin I by the aorta fragments pretreated with thiorphan (final concentration 3 *µ*M) from streptozotocin and saline treated animals (pg/mg of dry tissue; median [25th, 75th percentile]). *P* values are from Kruskal-Wallis nonparametric tests.

Analyte	Concentration in streptozotocin treated rats (*n* = 8), pg/mg of dry tissue; median [25th, 75th percentile]	Concentration in control rats (*n* = 7), pg/mg of dry tissue; median [25th, 75th percentile]	*P* value
Angiotensin I	912.83 [725.58, 1036.16]	627.72 [556.23, 659.39]	**0.06**
Angiotensin II	4.90 [3.63, 5.57]	3.73 [2.93, 4.19]	0.14
Angiotensin III	3.24 [2.47, 4.79]	2.63 [2.36, 3.38]	0.34
Angiotensin IV	1.04 [0.93, 1.44]	0.91 [0.68, 1.15]	0.14
Angiotensin (1–9)	2.81 [1.79, 4.67]	3.05 [1.76, 4.47]	0.95
Angiotensin (1–7)	2.51 [1.99, 3.17]	2.53 [1.85, 3.06]	0.95
Angiotensin (1–5)	1.41 [0.86, 1.55]	0.97 [0.65, 1.22]	0.14

**Table 4:** Ratios of concentrations of the products of classical and alternative pathways of exogenous ANG I (1 *µ*M) metabolism by aorta fragments of streptozotocin and saline treated animals (median [25th, 75th percentile]). *P* values are from Kruskal-Wallis nonparametric tests.

Ratio	Streptozotocin treated rats (*n* = 8); median [25th, 75th percentile]	Control rats (*n* = 7); median [25th, 75th percentile]	*P* value
No pretreatment			
ANG II/ANG (1–9)	3.98 [2.64, 4.96]	2.03 [1.50, 8.37]	0.22
ANG II/ANG (1–7)	4.90 [3.96, 7.33]	4.16 [1.93, 5.02]	0.13
ANG III/ANG (1–9)	1.20 [1.02, 1.67]	0.83 [0.53, 2.34]	0.22
ANG III/ANG (1–7)	1.63 [1.42, 1.94]	1.29 [1.15, 1.47]	**0.03**
ANG IV/ANG (1–9)	0.44 [0.23, 0.52]	0.34 [0.27, 0.61]	0.85
ANG IV/ANG (1–7)	0.50 [0.33, 0.70]	0.56 [0.37, 0.85]	0.49

Perindoprilat pretreatment			
ANG II/ANG (1–9)	0.87 [0.64, 1.07]	0.43 [0.30, 0.81]	0.08
ANG II/ANG (1–7)	1.03 [0.94, 1.09]	0.72 [0.70, 0.95]	0.06
ANG III/ANG (1–9)	1.00 [0.73, 1.11]	0.45 [0.37, 1.05]	0.08
ANG III/ANG (1–7)	1.18 [1.08, 1.34]	0.92 [0.90, 1.16]	**0.02**
ANG IV/ANG (1–9)	0.37 [0.29, 0.50]	0.16 [0.12, 0.36]	**0.04**
ANG IV/ANG (1–7)	0.50 [0.36, 0.53]	0.34 [0.24, 0.36]	**0.02**

Thiorphan pretreatment			
ANG II/ANG (1–9)	1.50 [0.99, 1.99]	1.22 [0.66, 1.84]	0.41
ANG II/ANG (1–7)	2.23 [1.69, 2.32]	1.31 [1.10, 2.00]	0.08
ANG III/ANG (1–9)	1.15 [0.80, 2.39]	1.03 [0.76, 1.29]	0.28
ANG III/ANG (1–7)	1.40 [1.29, 1.77]	0.98 [0.97, 1.20]	**0.02**
ANG IV/ANG (1–9)	0.43 [0.24, 0.52]	0.30 [0.15, 0.53]	0.65
ANG IV/ANG (1–7)	0.45 [0.35, 0.67]	0.35 [0.27, 0.47]	0.14

**Table 5:** Ratios of concentrations of the products of classical pathway of exogenous ANG I (1 *µ*M) metabolism by aorta fragments of streptozotocin and saline treated animals (median [25th, 75th percentile]). *P* values are from Kruskal-Wallis nonparametric tests.

Ratio	Streptozotocin treated rats (*n* = 8); median [25th, 75th percentile]	Control rats (*n* = 7); median [25th, 75th percentile]	*P* value
ANG IV/ANG II	0.09 [0.07, 0.12]	0.17 [0.14, 0.20]	**0.01**
ANG III/ANG II	0.32 [0.27, 0.41]	0.34 [0.28, 0.60]	0.56
ANG IV/ANG III	0.28 [0.22, 0.39]	0.49 [0.29, 0.56]	**0.049**

## References

[1] Cooper M. E. (2004). The role of the renin-angiotensin-aldosterone system in diabetes and its vascular complications. *American Journal of Hypertension*.

[2] Pernomian L., Pernomian L., Restini C. B. A. (2014). Counter-regulatory effects played by the ACE—Ang II—AT1 and ACE2—Ang-(1-7)—Mas axes on the reactive oxygen species-mediated control of vascular function: perspectives to pharmacological approaches in controlling vascular complications. *Vasa*.

[3] Vauquelin G., Michotte Y., Smolders I. (2002). Cellular targets for angiotensin II fragments: pharmacological and molecular evidence. *Journal of the Renin-Angiotensin-Aldosterone System*.

[4] Elsner M., Guldbakke B., Tiedge M., Munday R., Lenzen S. (2000). Relative importance of transport and alkylation for pancreatic beta-cell toxicity of streptozotocin. *Diabetologia*.

[5] Romero-Nava R., Rodriguez J. E., Reséndiz-Albor A. A. (2016). Changes in protein and gene expression of angiotensin II receptors (AT1 and AT2) in aorta of diabetic and hypertensive rats. *Clinical and Experimental Hypertension*.

[6] Campbell D. J., Kelly D. J., Wilkinson-Berka J. L., Cooper M. E., Skinner S. L. (1999). Increased bradykinin and ‘normal’ angiotensin peptide levels in diabetic Sprague-Dawley and transgenic (mRen-2)27 rats. *Kidney International*.

[7] Van Linthout S., Spillmann F., Lorenz M. (2009). Vascular-protective effects of high-density lipoprotein include the downregulation of the angiotensin II type 1 receptor. *Hypertension*.

[8] Failli P., Alfarano C., Franchi-Micheli S. (2009). Losartan counteracts the hyper-reactivity to angiotensin II and ROCK1 over-activation in aortas isolated from streptozotocin-injected diabetic rats. *Cardiovascular Diabetology*.

[9] Fraga-Silva R. A., Costa-Fraga F. P., Murça T. M. (2013). Angiotensin-converting enzyme 2 activation improves endothelial function. *Hypertension*.

[10] Bujak-Gizycka B., Madej J., Wołkow P. P. (2007). Measurement of angiotensin metabolites in organ bath and cell culture experiments by Liquid Chromatography - Electrospray Ionization—Mass Spectrometry (LC-ESI-MS). *Journal of Physiology and Pharmacology*.

[11] Olszanecki R., Bujak-Gizycka B., Madej J. (2008). Kaempferol, but not resveratrol inhibits angiotensin converting enzyme. *Journal of Physiology and Pharmacology*.

[12] Ladeia A. M., Sampaio R. R., Hita M. C., Adan L. F. (2014). Prognostic value of endothelial dysfunction in type 1 diabetes mellitus. *World Journal of Diabetes*.

[13] McFarlane R., McCredie R. J., Bonney M.-A. (1999). Angiotensin converting enzyme inhibition and arterial endothelial function in adults with Type 1 diabetes mellitus. *Diabetic Medicine*.

[14] Maser R. E., Lenhard M. J. (2003). Effect of treatment with losartan on cardiovascular autonomic and large sensory nerve fiber function in individuals with diabetes mellitus: a 1-year randomized, controlled trial. *Journal of Diabetes and its Complications*.

[15] Müller M., Fasching P., Schmid R., Burgdorff T., Waldhäusl W., Eichler H. G. (1997). Inhibition of paracrine angiotensin-converting enzyme in vivo: effects on interstitial glucose and lactate concentrations in human skeletal muscle. *European Journal of Clinical Investigation*.

[16] Lupi R., Del Guerra S., Bugliani M. (2006). The direct effects of the angiotensin-converting enzyme inhibitors, zofenoprilat and enalaprilat, on isolated human pancreatic islets. *European Journal of Endocrinology*.

[17] Furuhashi M., Ura N., Takizawa H. (2004). Blockade of the renin-angiotensin system decreases adipocyte size with improvement in insulin sensitivity. *Journal of Hypertension*.

[18] Frossard M., Joukhadar C., Steffen G., Schmid R., Eichler H. G., Müller M. (2000). Paracrine effects of angiotensin-converting-enzyme- and angiotensin-II- receptor-inhibition on transcapillary glucose transport in humans. *Life Sciences*.

[19] Folli F., Saad M. J. A., Velloso L. (1999). Crosstalk between insulin and angiotensin II signalling systems. *Experimental and Clinical Endocrinology and Diabetes*.

[20] Brosnihan K. B., Li P., Ferrario C. M. (1996). Angiotensin-(1-7) dilates canine coronary arteries through kinins and nitric oxide. *Hypertension*.

[21] Kahraman S., Aydin C., Elpek G. O., Dirice E., Sanlioglu A. D. (2015). Diabetes-resistant NOR mice are more severely affected by streptozotocin compared to the diabetes-prone NOD Mice: correlations with liver and kidney GLUT2 Expressions. *Journal of Diabetes Research*.

[22] Head R. J., Longhurst P. A., Panek R. L., Stitzel R. E. (1987). A contrasting effect of the diabetic state upon the contractile responses of aortic preparations from the rat and rabbit. *British Journal of Pharmacology*.

[23] Hong E., Villafaña S. (2009). Early diabetes in WKY and SHR produces decrease of the responses to angiotensin II and 5-HT and changes in the NO-GMPc pathway. *Clinical and Experimental Hypertension*.

[24] Arun K. H. S., Kaul C. L., Ramarao P. (2004). High glucose concentration augments angiotensin II mediated contraction via AT1 receptors in rat thoracic aorta. *Pharmacological Research*.

[25] Crespo M. J., Dunbar D. C. (2003). Enalapril improves vascular and cardiac function in streptozotocin-diabetic rats. *Cellular and Molecular Biology*.

[26] Fiordaliso F., Cuccovillo I., Bianchi R. (2006). Cardiovascular oxidative stress is reduced by an ACE inhibitor in a rat model of streptozotocin-induced diabetes. *Life Sciences*.

[27] Mahmood A., Jackman H. L., Teplitz L., Igić R. (2002). Metabolism of angiotensin I in the coronary circulation of normal and diabetic rats. *Peptides*.

[28] Verma A., Shan Z., Lei B. (2012). ACE2 and Ang-(1–7) confer protection against development of diabetic retinopathy. *Molecular Therapy*.

[29] Patel V. B., Parajuli N., Oudit G. Y. (2014). Role of angiotensin-converting enzyme 2 (ACE2) in diabetic cardiovascular complications. *Clinical Science*.

[30] Vinh A., Widdop R. E., Drummond G. R., Gaspari T. A. (2008). Chronic angiotensin IV treatment reverses endothelial dysfunction in ApoE-deficient mice. *Cardiovascular Research*.

[31] Vinh A., Widdop R. E., Chai S. Y., Gaspari T. A. (2008). Angiotensin IV-evoked vasoprotection is conserved in advanced atheroma. *Atherosclerosis*.

[32] Nasser M., Clere N., Botelle L. (2014). Opposite effects of angiotensins receptors type 2 and type 4 on streptozotocin induced diabetes vascular alterations in mice. *Cardiovascular Diabetology*.

[33] Karnik S. S., Unal H., Kemp J. R. (2015). Angiotensin receptors: Interpreters of pathophysiological angiotensinergic stimulis. *Pharmacological Reviews*.

